# Enhanced osteogenesis of quasi-three-dimensional hierarchical topography

**DOI:** 10.1186/s12951-019-0536-5

**Published:** 2019-10-03

**Authors:** Mengfei Yu, Yu Liu, Xiaowen Yu, Jianhua Li, Wenquan Zhao, Ji’an Hu, Kui Cheng, Wenjian Weng, Bin Zhang, Huiming Wang, Lingqing Dong

**Affiliations:** 1 The Affiliated Stomatologic Hospital, School of Medicine, Zhejiang University, Hangzhou, 310003 China; 2 The State Key Laboratory of Fluid Power Transmission and Control, Zhejiang University, Hangzhou, 310027 China; 3 School of Materials Science and Engineering, State Key Laboratory of Silicon Materials, Zhejiang University, Hangzhou, 310027 China; 4 Hangzhou Dental Hospital, Hangzhou, 310006 China

**Keywords:** Osteogenesis, Quasi-three-dimensional, Hierarchical, Nanorods, Titania

## Abstract

Natural extracellular matrices (ECMs) are three-dimensional (3D) and multi-scale hierarchical structure. However, coatings used as ECM-mimicking structures for osteogenesis are typically two-dimensional or single-scaled. Here, we design a distinct quasi-three-dimensional hierarchical topography integrated of density-controlled titania nanodots and nanorods. We find cellular pseudopods preferred to anchor deeply across the distinct 3D topography, dependently of the relative density of nanorods, which promote the osteogenic differentiation of osteoblast but not the viability of fibroblast. The in vivo experimental results further indicate that the new bone formation, the relative bone-implant contact as well as the push-put strength, are significantly enhanced on the 3D hierarchical topography. We also show that the exposures of HFN7.1 and mAb1937 critical functional motifs of fibronectin for cellular anchorage are up-regulated on the 3D hierarchical topography, which might synergistically promote the osteogenesis. Our findings suggest the multi-dimensions and multi-scales as vital characteristic of cell-ECM interactions and as an important design parameter for bone implant coatings.

## Introduction

Three-dimensional (3D) natural extracellular matrix (ECM) feature has inspired people to construct physiological-mimicking artificial environments for cellular culture [1]. As compared to the two-dimensional system, cells cultured in 3D environments are able to growth and contact in three dimensions thus reveal striking differences in physiological processes such as adhesion, proliferation, migration and differentiation [2]. Therefore, a wide variety of materials such as collagen, gel/hydrogel, alginate etc. have been used to engineer 3D matrices [3–6]. However, the features of soft tissue-like stiffness and biodegradability restrict their applications as stable coatings of implant for osteogenesis. Although the additive manufacturing technology has been extensively used to engineer various metal and ceramic 3D matrix composites [7], it still remains a challenge to construct desired 3D or quasi-3D nanoscaled topography [8].

Additionally, ECM shows a hierarchical organization ranging from nano to macron scales. The biomimetic nano-to-microscaled hierarchical topography features have demonstrated distinct biological activities [9]. For example, the hierarchical hybrid micro/nano-textured surface topography with titania nanotubes has been demonstrated to enhance multiple osteoblast functions [10]. Micropit and nanonodule hybrid topography of TiO_2_ also showed to up-regulate the proliferation and differentiation of osteoblast [11]. However, it is still unclear of the mechanism underlying how cells response to the micro/nano hierarchical topography due to the cellular sensitivity to various material features (such as topography, hardness, hydrophilicity, etc.) as well as the lake of effective topography control under the same processing [12, 13].

In this study, we demonstrated the enhanced osteogenesis of quasi-three-dimensional hierarchical topography composed of density-controlled titania nanodots and nanorods, which was prepared by using spin self-assembly coating process and subsequent hydrothermal treatment. The in vitro osteogenic performance was evaluated by exploring the adhesion, proliferation and differentiation of osteoblast as well as the viability of fibroblast. And the in vivo osteointegration performance was evaluated by using histological analysis and push-out mechanical strength test after 4 weeks or 8 weeks of implantation. Furthermore, the possible mechanism underlying cells response to the quasi-3D hierarchical topography based on the initial adsorption behaviors of fibronectin was also proposed.

## Materials and methods

### Construction of quasi-three-dimensional hierarchical topography

The quasi-three-dimensional hierarchical topography composed of titania nanodots and nanorods were engineered by two steps, as we described previously [14]. Briefly, the nanodots coating was firstly prepared by using the phase-separation-induced self-assembly technology. An ethanol solution with a molar ratio of AcAc/TBOT/H_2_O of 0.3:1:1 with 40 mg/L PVP was dropped onto Si or SiO_2_ substrate by spin-coating at 8000 rpm for 40 s. The spin-coated substrate was then heated at 500 °C for 2 h in a muffle furnace. The diameter of obtained nanodots was ranged from 30 to 110 nm, and the density was about 5.6 × 10^10^/cm^2^.

Then, the nanorods films were further grown on the obtained nanodots films by using the hydrothermal treatment. Briefly, 30 mL of concentrated HCl (36.5–38% by weight) was mixed with 30 mL of deionized water and the designed amount of TBOT to obtain a total volume of 60 mL. During the preparing hydrothermal solutions, HCl with water for dilution was stirred under ambient condition for 5 min. Then a designed amount of TBOT was added and stirred until becoming a clear solution. The hydrothermal solution was transferred to a 50 μL Teflon-lined stainless steel autoclave. The hydrothermal growth was conducted at 160 °C for 2 h in an electric oven. The dimension and density of nanorods were controlled by the adjustment of the concentration of TBOT. The clinical used titanium implant was also used as the control. The surface morphology of these samples was examined by field-emission scanning electron microscopy (FESEM, SU-70, Hitachi). The diameter and height of nanorods were qualitative analyzed of more than 100 nanorods based on the SEM observation results (n ≥ 10). The density of nanorods and percentage of nanorods area were calculated as the number and occupied area of nanorods on every square micron, respectively (n ≥ 10), based on the SEM images.

### Cell culture

Mouse calvaria-derived, pre-osteoblastic MC3T3-E1 cells (CRL-2594, ATCC) were used as model cells. Cells were cultured in alpha-modified Minimum Essential Medium (Gibco), supplemented with 10% fetal bovine serum (PAA), 1% sodium pyruvate (Gibco), 1% antibiotic solution containing 10,000 units/mL penicillin and 10,000 mg/mL streptomycin (Gibco), 1% MEM non-essential amino acids (Gibco) in a humidified atmosphere of 5% CO_2_ at 37 °C. Subconfluent MC3T3-E1 on polystyrene dishes were trypsinized with 0.25% trypsin/1 mM EDTA (Gibco) and were subcultured on the substrates at a density of 4 × 10^4^ cells/cm^2^. The NIH3T3 fibroblasts, cultured in Dulbecco’s modified Eagle medium (PAA) and supplemented with 10% fetal bovine serum and antibiotic solution, were also inoculated onto substrates above. The culture medium was renewed every 2 days for both cell types.

### Morphology and morphometry of cells

In order to evaluate the initial attachment behaviors of cells on various substrates, MC3T3-E1 cell morphology was observed by SEM. After 3 h, culture media were removed and the specimens were fixed with 2.5% glutaraldehyde in PBS (0.1 M, pH 7.0) for more than 4 h, and washed three times in PBS for 15 min respectively. Then they were post-fixed with 1% OsO_4_ in PBS for 1 h and washed three times in PBS for 15 min, followed by dehydration by a graded series of ethanol (50%, 70%, 80%, 90% and 95%) for 15 min and by 100% ethanol twice for 20 min. Afterwards, the specimens were transferred to the mixture of ethanol and isoamyl acetate (v:v = 1:1) for 30 min and pure isoamyl acetate for 1 h, followed by final dehydration in Hitachi Model Hcp-2 critical point dryer with liquid CO_2_. Confocal laser scanning microscopy (LSM510 META, Zeiss) was used to examine cytoskeletal arrangement in MC3T3-E1 cell seeded onto specimens with different surfaces. After 3 h of culture, cells were fixed in 4% paraformaldehyde and immunochemically stained with goat anti-actin polyclonal antibody (SC-1615, Santa Cruz Biotechnology), followed by a DylightTM 594-conjugated anti-goat secondary antibody (Jackson), counter stained with Hoechst 33258 (Sigma Aldrich), respectively. The area, perimeter, Feret’s diameter and migration of cells were quantified using Image-Pro Plus.

### Cell attachment and proliferation assays

Initial attachment of MC3T3-E1 and fibroblasts to specimen substrates (1 × 1 cm^2^) was evaluated by measuring the amount of cells attached to samples after 6 and 24 h of incubation. Further cell proliferation was quantified in terms of cell density for 2 and 5 culture days. 500 μL cell suspension with a density of 4 × 10^4^ cells/cm^2^ was inoculated to a 24-multiwell plate containing the samples with different surfaces. After 6 and 24 h, samples were transferred to another 24-well after washed by PBS three times, while there was no need to wash the samples when taking proliferation assays, then 500 μL fresh culture media and 50 μL of MTS (G358B, Promega, USA) solution were added in each well for 4 h at 37 °C. Finally at indicated time, 120 μL of the culture media was dispensed onto another 96-mutliwell plate, and colorimetric measurement of formazan dye was performed on a microplate reader at 490 nm.

### Western blot analysis

MC3T3-E1 cells were cultured on the substrates a density of 4 × 10^4^ cells/cm^2^ for 5, 7 and 14 days. Cells on each type of specimen were treated with cell lysis buffer (9803, Cell Signaling). The proteins were collected and separated by sodium dodecyl sulfate-polyacrylamide gel electrophoresis (SDS-PAGE) on 5% Tris–HCl reducing gels, followed by transferring to the PVDF membrane (Millipore). The membrane was blocked by incubating in a blocking solution containing 5% skim milk (BD) and 0.1% TWEEN 20 (Fluka Chemika) for 2 h. After that, the sheets were incubated with ALP (ab65834, Abcam) or COL-I (ab6308, Abcam) at 4 °C overnight. Then HRP-anti-Rb antibody (050884, KPL) was used for the expression of ALP and COL-I. Band densities on the Western blots were assessed using the Quantity One software (Versa Doc 5000, BioRad) and normalized to GAPDH proteins.

### Gene expression analysis

Gene expression was analyzed by real-time reverse transcription-polymerase chain reaction (real-time RT-PCR). Total RNA was extracted from cultured MC3T3-E1 cells on the substrates with a density of 4 × 10^4^ cells/cm^2^ using the TRIzol reagent (Invitrogen) according to the manufacturer’s recommended protocol. The concentration and purity of total RNA were calculated with the absorbance at 260 and 280 nm. Real-time PCR was performed in the LightCycler using SYBR green detection. Gene expression was normalized to the housekeeping gene GAPDH. PCR products were subjected to a melting curve analysis and analyzed by the 2^−∆∆CT^ method.

### Bone nodule visualization

Bone nodule formation of the cells that cultured on different coatings was observed by SEM. The cells were cultured in the presence of 5 mM glycerol-2-phosphate disodium salt hydrate (β-glycerophosphate; Sigma) and 50 μg/mL l-ascorbic acid (Sigma) when became confluency with an initial density of 4 × 10^4^ cells/cm^2^. After 21 days, the specimen was dehydrated in an ethanol solution of varying concentrations (i.e., 30, 50, 70, 90, and 100% twice, respectively) for 2 min at each concentration after being fixed with 2.5% glutaraldehyde in PBS (0.1 M, pH 7.0) overnight at 4 °C. It was then dried in 100% hexamethyldisilazane (HMDS, Sigma) for 5 min and later dried in air after the removal of HMDS. After being completely dried, the specimen was coated with gold, and observed by SEM. The Ca elemental analysis of calcium nodule was carried out on the FE-SEM (Hitachi SU-70) equipped with an energy dispersive X-ray spectrum (EDS, Inca Energy-200) at an accelerating voltage of 10 kV.

### Collagen production

Sirius red staining-based colorimetric assay was employed to quantify collagen production in fibroblasts. Fibroblasts cultured on the substrates with a density of 4 × 10^4^ cells/cm^2^ were washed twice with PBS and fixed with Bouin’s fluid for 1 h at room temperature, which was prepared by mixing 15 mL saturated aqueous picric acid with 5 mL 35% formaldehyde and 1 mL glacial acetic acid. The cultures were washed by running tap water for 15 min before staining with 1 mL saturated solution of picric acid containing 0.04% of Fast green and 0.1% of Sirius red F3B, and incubated in the dark for 1 h with mild shaking. They were then rinsed several times with distilled water until the elution fluid was completely free of color. After that, the samples were dissolved in 0.25 mL 0.1 N sodium hydroxide using a microplate shaker for 30 min at room temperature. Then 120 μL of the liquids were transferred to Nunc microtiter plates and the optical densities were measured using a spectrophotometer at 630 nm and 450 nm, respectively. Results are presented relative to protein.

### Cell adhesion assay

The adhesive strength of MC3T3-E1 cells and fibroblasts to on the substrates was evaluated by the percentage of remaining cells after enzymatic treatment. Cells incubated on samples with a density of 4 × 10^4^ cells/cm^2^ for 24 h were rinsed once with PBS to remove non-adherent cells, and then detached from the substrates by treatment with 0.05% v/v trypsin–EDTA at 37 °C for 1 min. The detached and remaining cells were detected by MTS assay.

### Protein adsorption assay

Bovine serum albumin (Pierce Biotechnology) was used as a model protein. 250 μL of protein solution (1 μg/mL protein/saline) was pipetted into a 24-multiwell plate with substrates (1 × 1 cm^2^). After incubation for 2 and 24 h at 37 °C, non-adherent proteins were removed and mixed with bicinchoninic acid (Pierce Biotechnology) at 37 °C for 30 min. The amount of the removed albumin as well as the total amount of albumin inoculated was quantified using a microplate reader at 570 nm. The rate of albumin adsorption was calculated as the percentage of albumin adsorbed to relative to the total amount.

### Enzyme-linked immunosorbent assay

The substrates (1 × 1 cm^2^) were immersed in excess BSA solution for 24 h at 37 °C for BSA adsorption. Next, all the samples were immersed in a solution of excess fibronectin (Fn) (10 μg/mL from human plasma, Sigma-Aldrich) for another 24 h at 37 °C for Fn adsorption. After that, surfaces were rinsed with PBS and non-specific antibody binding was blocked using a blocking buffer (1% BSA/PBS) for 30 min at 37 °C. The primary monoclonal antibody HFN7.1 (Developmental Studies Hybridoma Bank) directed against the flexible linker between the 9th and 10th type III repeat and mAb1937 (Millipore) directed against the 8th type III repeat were used. Substrates were incubated with primary antibody (1:4000 for HFN7.1 and 1:1000 mAb1937 in blocking buffer) for 1 h at 37 °C. After washing with 0.1% Tween 20, all samples were incubated with alkaline phosphatase-conjugated anti-mouse IgG (1:5000, Jackson ImmunoResearch Laboratories) for 1 h at 37 °C, then washed again, and incubated with 4-methylumbelliferyl phosphate (4-MUP) liquid substrate system (Sigma-Aldrich) for 45 min at 37 °C. Reaction products were quantified using a fluorescence plate reader at 365 nm excitation/460 nm emission.

### Hemolysis test

Rabbit blood (8 mL) anti-coagulated with potassium oxalate was diluted with saline (10 mL). 0.2 mL of the blood was added to 10 mL of saline containing Ti as well as M-NR in different test tubes. A positive (100% hemolysis induced by replacing saline with distilled water) and negative (0% hemolysis, saline with no material added) were also set up. Each set of experiments was done in duplicate. All test tubes were incubated at 37 °C for 60 min. After incubation, the tubes were centrifuged at 750*g* for 5 min. The percentage of hemolysis was calculated by measuring the optical density of the supernatant solution at 540 nm in a UV–visible spectrophotometer as per the following formula:

$$ Hemolysis     \, \left ( \% \right) = \frac{{{\text{OD}}\;{\text{of}}\;{\text{the}}\;{\text{test}}\;{\text{sample}} - {\text{OD}}\;{\text{of}}\;{\text{the}}\;{\text{negetive}}\;{\text{control}}}}{{{\text{OD}}\;{\text{of}}\;{\text{the}}\;{\text{postive}}\;{\text{control}} - {\text{OD}}\;{\text{of }}\;{\text{the}}\;{\text{negetive}}\;{\text{control}}}} \times 100{\text{\% }} $$

### Implant biomechanical push-out test

The implant biomechanical push-out test was used to assess the biomechanical strength of bone-implant integration. Briefly, 12 male New Zealand white rabbits weighing 2.5–3.0 kg were used in this study. The animals were anesthetized intramuscularly with SuMianXin II (0.1–0.2 mL/kg, Quartermaster University of PLA, Chang Chun, P. R. of China, The Military Veterinary Institute). Lidocaine was injected locally into the surgical site before the operation. The medial surfaces of proximal tibias were selected for implantation by drilling with a 0.8 mm round burr and then enlarged using reamers with a final drill diameter of 3.0 mm. All drilling procedures were carried out under profuse sterile saline irrigation. The titanium implants (diameter, 3 mm; length, 8 mm) and the substrates were inserted into each prepared hole per tibia with 2 mm left outside. Tibias containing a cylindrical implant were harvested at week 4 and 8 of healing with the top surface of the implant being horizontal. Spiral CT (Brilliance 64 ct, Philips) was used to confirm that implants were free from cortical bone support to the lateral and bottom sides of the implant. A testing machine (SANS CTT2500) equipped with a 2000 N load cell was used to load the implant vertically upward at a crosshead speed of 1 mm/min. The push-out value was determined by measuring the peak of the load–displacement curve. The experiment was performed in accordance with the guidelines for animal care established by Zhejiang University, Hangzhou, China.

### Histomorphometric analysis

Each substrate was inserted into anterior-distal surfaces of per femur with 2 mm left outside. The femurs with the implants were isolated after 4 and 8 weeks, and fixed in 4% paraformaldehyde solution. After decalcification, the implants were gently removed, and the femoral tissues were dehydrated, cleared and embedded in paraffin. Tissue sections, 4 μm in thickness, were mounted on glass slides and subjected to H&E staining. Images were taken under a Nikon Eclipse E600 microscope, and the newly formed bone area, which was restricted to the 0.5 mm area surrounding the implant, was measured with Spot Advanced Software (Diagnostic Instruments, Sterling Heights, MI, USA). The percentage of new bone edges in direct contact with the implant surface was also determined.

### Statistical analyses

The number of samples was 3 for all studies, except for cell morphometry (n = 9), and the experiments about implant in vivo (n = 6). All values are expressed as mean ± standard deviation. Statistical analyses were carried out by a one-way analysis of variance (one-way ANOVA) and Scheffe’s post hoc test with the SPSS software for multiple comparison tests, or using Student’s t-test. Differences were considered statistically significant when P < 0.05.

## Results

### Quasi-three-dimensional hierarchical topography

**Fig. 1:**
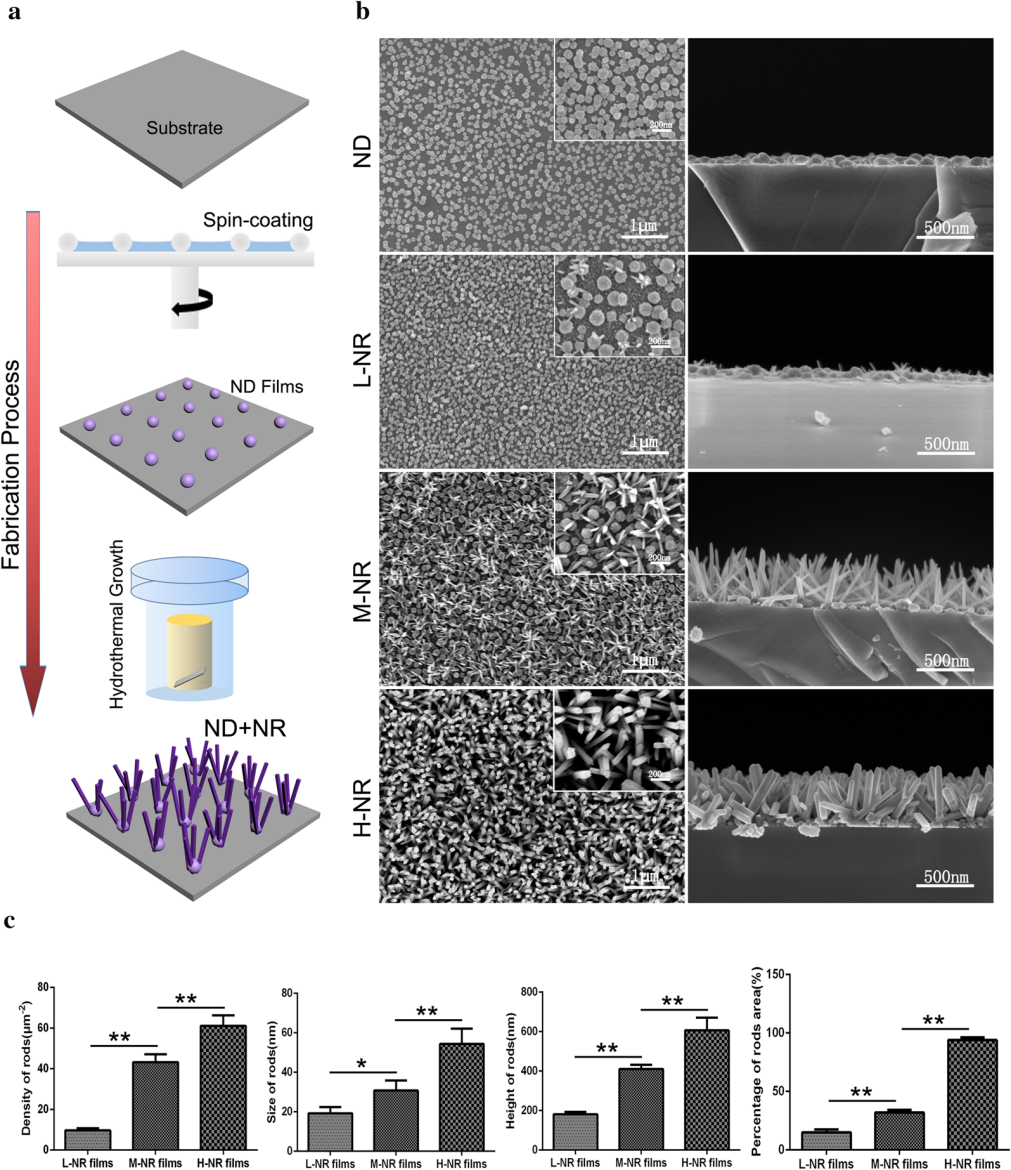
Quasi-three-dimensional hierarchical topography. **a** Schematic diagram of the fabrication process. **b** SEM images from top (right) and cross-sectional (left) view of the nanodots film and three typical quasi-three-dimensional hierarchically topographies with controlled density of nanorods. **c** Quantitative analysis of the density, diameter, height as well as the percent of nanorods area of the three topographies (n ≥ 10)

As shown in Fig. 1a, the nanodots film was firstly prepared by using the phase-separation-induced self-assembly technology. And the obtained nanodots film provided the crystal seeds for nanorods growth during the subsequent hydrothermal treatment. By controlling the concentrations of TBOT (Ti source), we strategized a series of size- and density-controlled rutile TiO_2_ nanorods coatings using the same processing. As demonstrated in the SEM images and the corresponding quantitatively analysis (Fig. 1b, c), we successfully achieved three typical quasi-three-dimensional hierarchically topographies: low density nanorods film (L-NR film) with 20 nm of dimension, 180 nm of height and 1000 nm of spacing, middle density nanorods film (M-NR film) with 30 nm of dimension, 420 nm of height and 300 nm of spacing, as well as the high density nanorods film (H-NR film) with 50 nm of dimension, 650 nm of height and 30 nm of spacing.

### Cellular morphology on various substrates

**Fig. 2:**
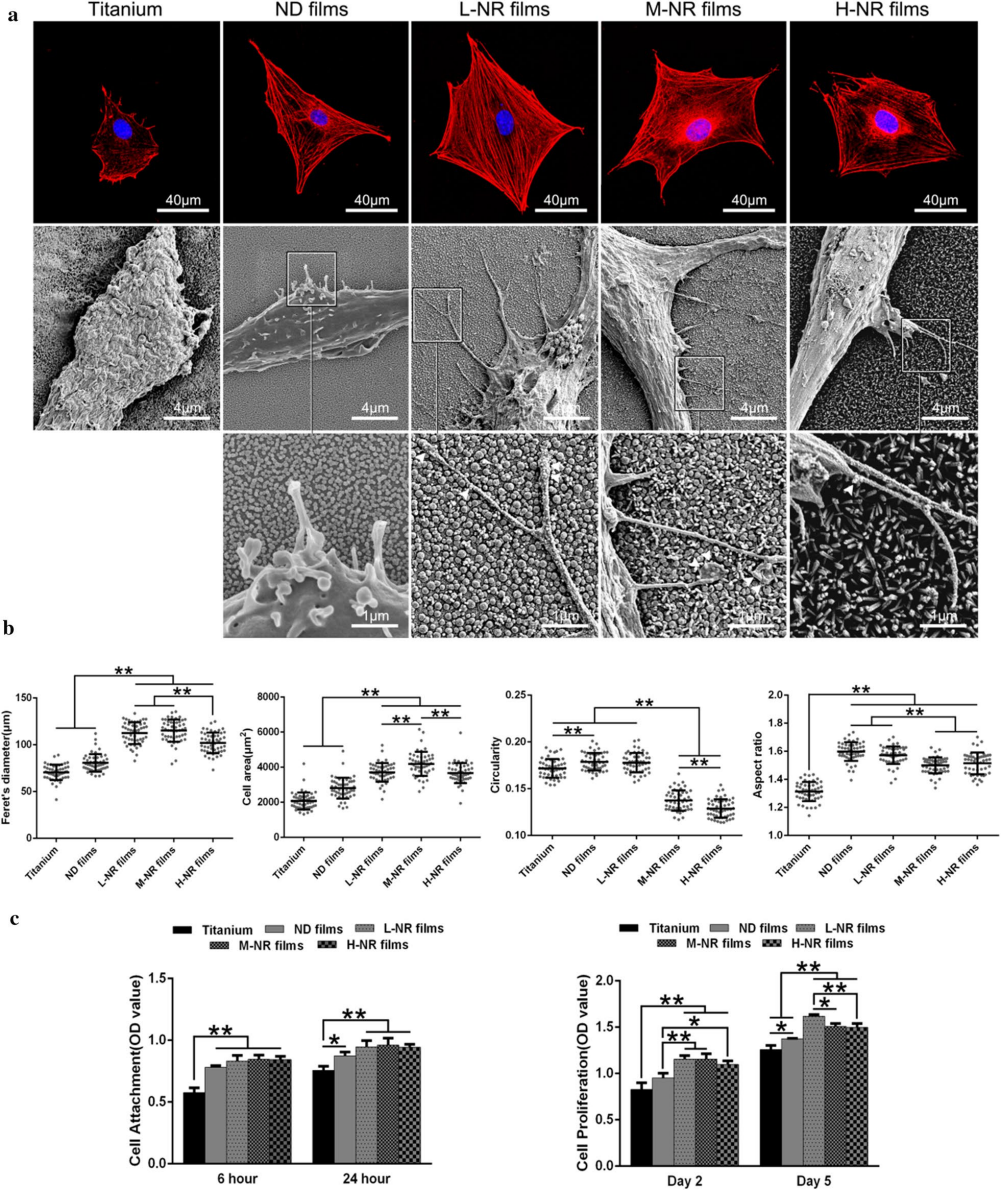
Cellular adhesion behavior on various substrates. **a** Upper, immunofluorescence observation of cellular cytoskeletonal morphology by staining the actin (red), and nuclei (blue). Middle, SEM observation of the typical cellular pseudopodia. Lower, the corresponding local amplification of filopodia (white arrows). **b** The corresponding quantitative analysis of cellular Feret’s dimeter, average cell area, circularity and aspect ratio. **c** Cellular viability analysis (CCK-8 assay) for cellular adhesion (6 and 24 h) and proliferation (2 and 5 days)

To observe the cellular spreading behavior on various substrates, we carried out immunostaining and SEM after 1 day of culture. As shown in Fig. 2, the cellular actin cytoskeleton on titanium substrate appeared to be relative round, while they revealed to be fully spreading and noticeable pseudopods extensions on various nanorods films (SEM images). Moreover, it’s worth noting that filopodia preferred to anchor on the nanorods, deeply across the distinct 3D topography. The corresponding quantitative analysis of cellular Feret’s dimeter, average cell area, further confirmed these results and suggested that cells on the M-NR films achieved the optimal spreading. Additionally, the decreased circularity and increased aspect ratio suggested greater protrusions and longer cell morphology.

### Cellular proliferation on various substrates

The cellular adhesion and proliferation behaviors were also evaluated to further study the effects of surface topography on cell behavior. As shown in Fig. 2c, the number of adhered cells on nanorods films was significantly up-regulated compared to that of titanium and nanodots films. Specifically, cells on the M-NR and L-NR groups exhibited the optimal adhesion and proliferation, respectively.

### In vitro evaluation of osteogenic differentiation of osteoblast

**Fig. 3:**
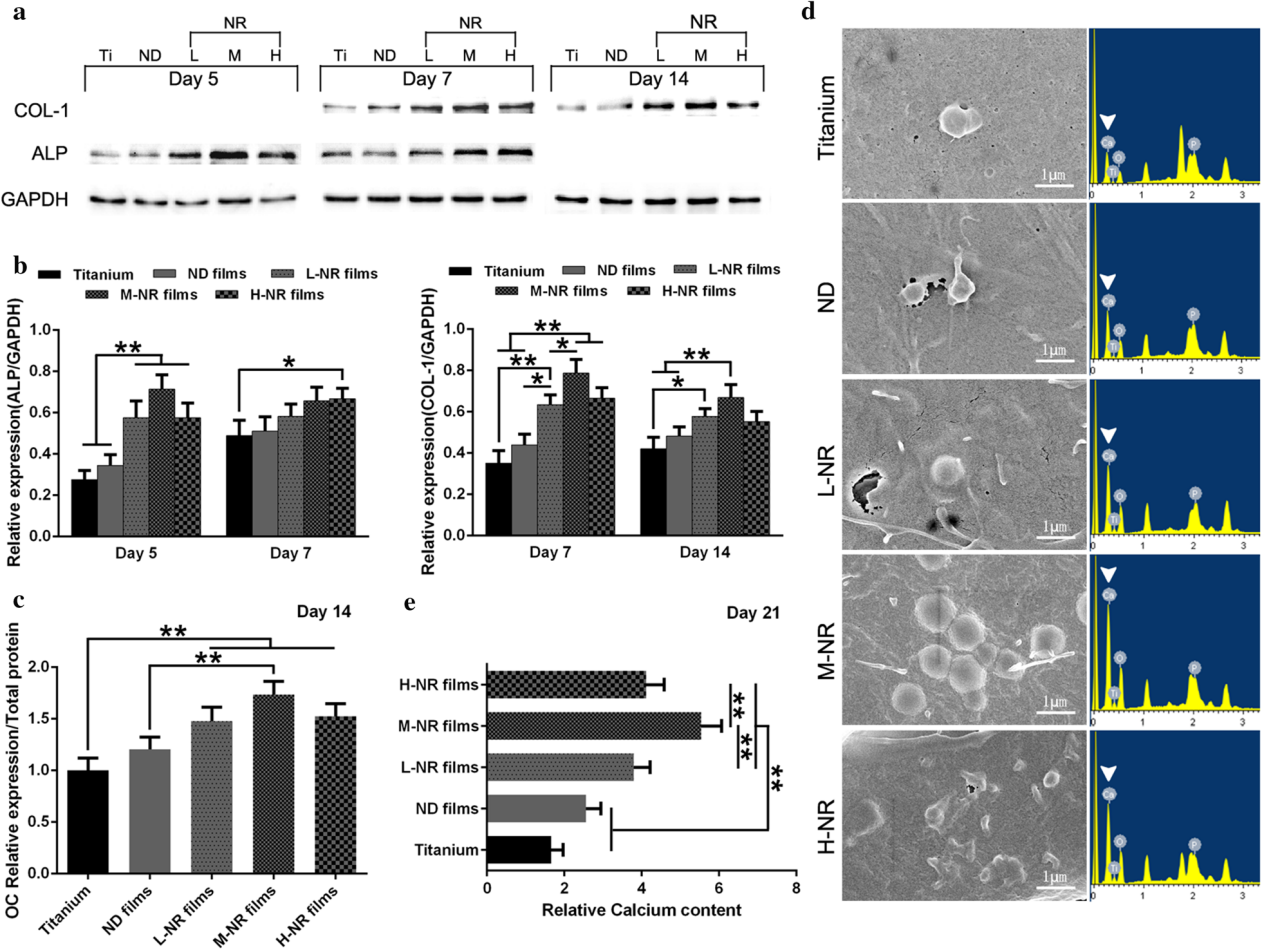
*In vitro* evaluation of osteogenic differentiation of osteoblast on various substrates. **a** Western blot analysis of relative protein expression of early and middle-stage osteogenic differentiation markers ALP and COL-I, respectively, after 5, 7 and 14 days of culture. And **b** the corresponding PCR results of relative mRNA expression. **c** ELISA assay analysis of relative protein expression of later osteogenic differentiation marker osteocalcin (OC) after 14 days of culture. **d** SEM observation (right) and the corresponding EDS Ca elemental analysis (left) of calcium nodule after 21 days of culture. **e** Analysis of the relative calcium content

To probe the osteogenic differentiation of osteoblast on various substrates, we carried out western blot (WB) analysis of relative protein expression of the early osteogenic differentiation markers ALP (day 5 and 7) and the middle-stage osteogenic differentiation markers COL-I (day 7 and 14), respectively. As shown in Fig. 3a, cellular expression level of ALP and COL-I on all nanorods films showed significantly up-regulated compared to that of titanium and nanodots groups. Moreover, cells on the M-NR films exhibited both up-regulated expression of ALP and COL-I upon various naorods films. The corresponding gene expression results of PCR the WB results also confirmed the WB results, as indicated in Fig. 3b. The relative protein expression of later osteogenic differentiation marker osteocalcin (OC) was also consistent with the above results (Fig. 3c). Moreover, cells on M-NR films still demonstrated significantly up-regulated number of calcium nodule and relative calcium content after 21 days of culture (Fig. 3d, e). Interestingly, the Ca:P ratio also showed a slight difference between the four groups: Ti (0.89 ± 0.18), ND (1.17 ± 0.14), L-NR (1.36 ± 0.22), M-ND (1.61 ± 0.24), H-ND (1.46 ± 0.16), respectively.

### Viability of fibroblast on various substrates

**Fig. 4:**
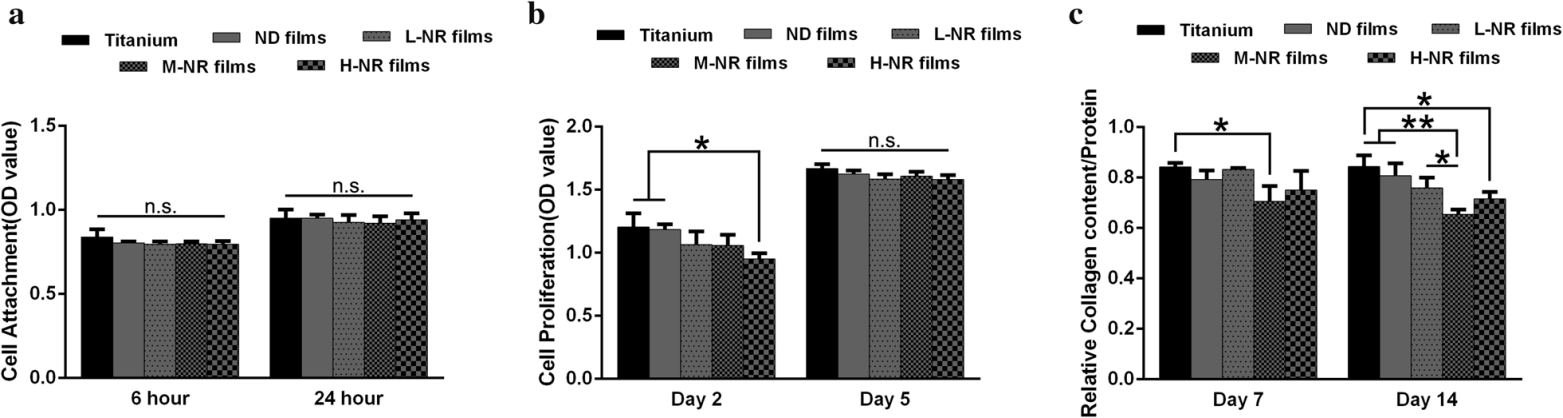
Evaluation of fibroblast viability on various substrates. Cellular viability analysis (CCK-8 assay) for **a** cellular adhesion and **b** proliferation. **c** Analysis of relative protein expression of COL-1 on various substrate after 7 and 14 days of culture

We further evaluated the adhesion, proliferation and collagen production behaviors of fibroblast on various substrates. As shown in Fig. 4a, there was no significantly difference on the adhesion behaviors between various substrates. In contrast, fibroblast exhibited up-regulated proliferation and collagen production on titanium substrate compared to other substrates. And the M-NR film appeared to be most disadvantageous to cellular proliferation and collagen production (Fig. 4b, c).

### Initial protein adsorption behaviors on various substrates

**Fig. 5:**
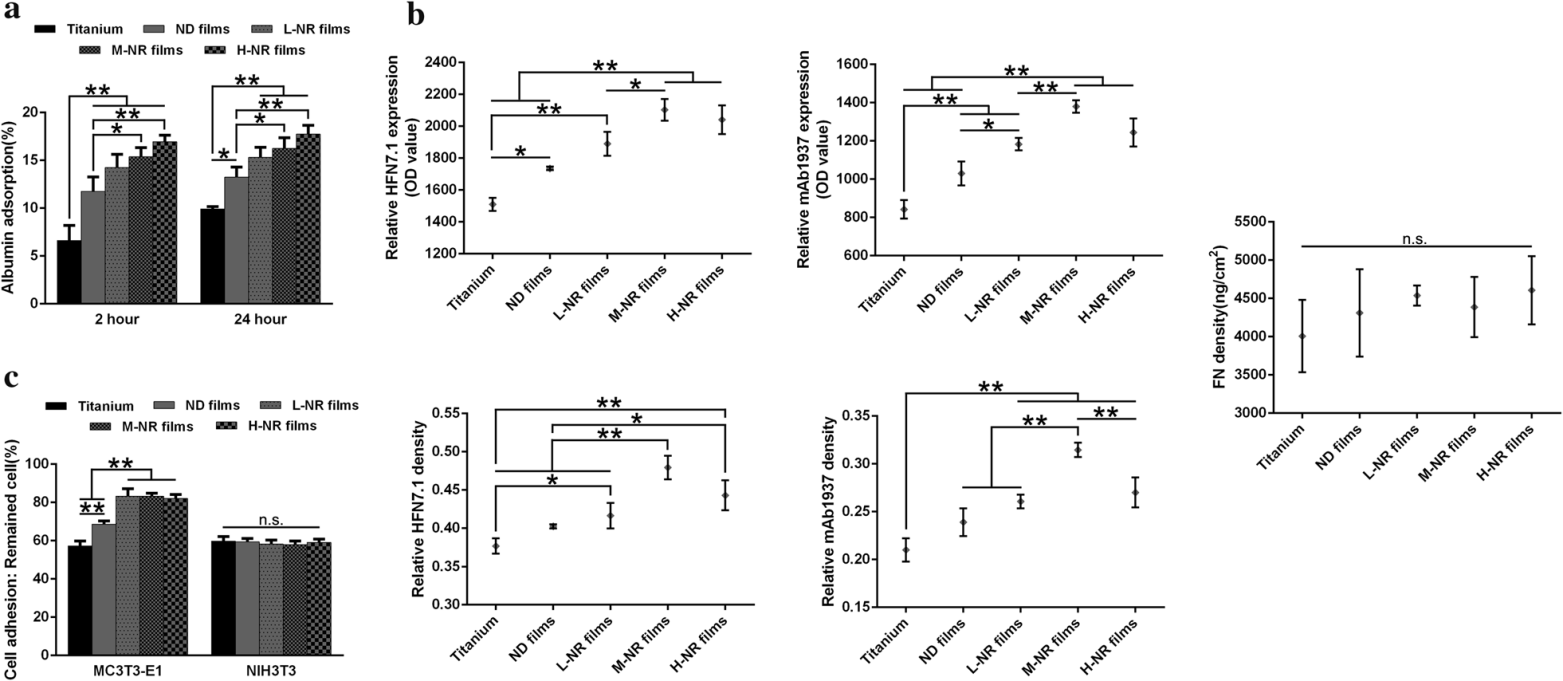
Initial protein adsorption behaviors and their effects on cellular adhesion on various substrates. **a** The amount of initial adsorption (2 and 24 h) of bovine serum albumin (BSA) protein. **b** Adsorption behavior of fibronectin (Fn): upper, the amount of exposure of HFN7.1 and mAb1937 critical functional motifs of Fn; middle, the amount of adsorbed Fn protein; lower, the relative exposure per Fn unit of HFN7.1 and mAb1937 critical functional motifs. **c** Analysis of cellular adhesion strength of osteoblast and fibroblast on various substrates after 24 h of culture

To gain insight into the mechanism underlying how the 3D hierarchically topography regulates the adhesion, proliferation and differentiation of osteoblast and fibroblast, we focused our attention on the initial protein adsorption behaviors on various substrates. As shown in Fig. 5a, the amount of initial adsorption of bovine serum albumin (BSA) protein increased along with the density of nanorods. However, these results were not consistent with the total exposure amount as well as the relative exposure per Fn unit of HFN7.1 and mAb1937 critical functional motifs. Although there was significantly difference on the total amount of initial Fn adsorption, we observed that the M-NR film group appeared to be more beneficial to the exposure of HFN7.1 and mAb1937 critical functional motifs (Fig. 5b). These distinct initial protein adsorption behaviors subsequently regulated the adhesion of osteoblast but had no significantly impact on fibroblast.

### In vivo osteointegration performance

**Fig. 6:**
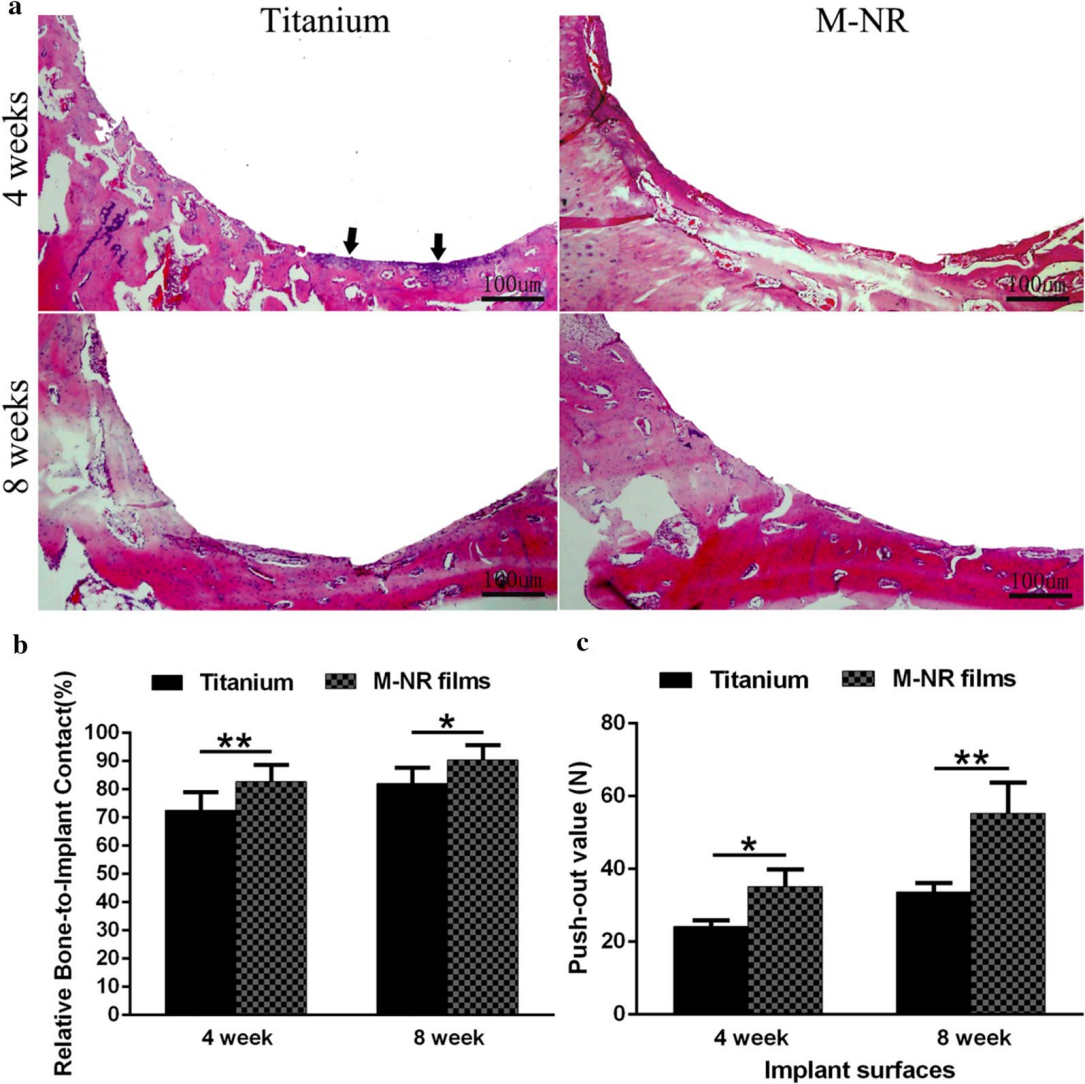
In vivo evaluation of osteointegration of the quasi-three-dimensional hierarchical topography. **a** Histologic analysis at the bone-implant interface of clinical titanium (control) and M-NR group after implantation for 4 and 8 weeks. The arrows indicate the inflammation regions. **b** The corresponding quantitative analysis of the relative bone-to-implant contact. **c** The evaluation of push-out strength (n ≥ 5)

We further evaluated the in vivo osteointegration performance of various substrates. Before that, we evaluated the bio-compatibility by the blood cell safety assays (data not shown). The results indicated that the Ti and M-NR film group had no negative effect on haemolysis, suggesting a good bio-compatibility. As shown in Fig. 6a, the histological observation of the bone-implant interface suggested that the distinct quasi-three-dimensional hierarchical topography enhanced the new bone formation after 4 and 8 weeks of implantation compared to that of the clinical titanium, further supported by the quantitative analysis of the relative bone-implant contact (Fig. 6b). In contrast, we even observed that there were some inflammation regions on the boundary between clinical titanium and host bone after 4 weeks of implantation (Fig. 6a). Moreover, the push-out strength evaluation further confirmed the tight and novel osseointegrated performance of quasi-three-dimensional hierarchical topography, indicating nearly 50% superiority in push-out strength compared to that of the clinical titanium (Fig. 6c). To further evaluate the mechanical stability of surface nanostructures upon implantation, we further observed the surface topography of the implant after push-out test followed by ultrasonic cleaning for 1 h (data not shown). The results indicated that the prepared surface nanorods structures were maintained without obvious damage in this study. We speculated that there results might be attributed to mechanical stability originated from the in situ growth process.

## Discussion

Fabrication of material features mimicking natural cellular ECMs provides physiologically relevant cellular microenvironments and thus has achieved distinct biological activities [15]. Although materials with 3D hierarchical structure features have been extensively studied, most of them are still limited to soft tissue scaffolds [16, 17]. In terms of the bioactive coating materials used in hard tissue engineering, good chemical stability and mechanical strength are required. Therefore, the bioactive inorganic material TiO_2_ was selected here to construct the quasi-three-dimensional hierarchical topography [18, 19]. And the in situ growth processing of TiO_2_ nanorods ensured the stability of the coating, which was confirmed in our previous work [14]. Moreover, the distinct nanorods feature might act as a “nailing” effect between the coating and host bone, which was beneficial to the osteointegration performance.

The density of nanorods was observed to be critical to the cellular responses on various substrates. We here speculate that cells are able to sense the spatial scale of a comparable size to cells themselves. While the density of nanorods was too low or high (L-NR and H-NR groups), cells were still inclined to perceive two-dimensional microenvironments. In contrast, when the density is moderate (M-NR), osteoblasts will be able to fully sense the quasi-three-dimensional hierarchical topography and thus appear to be significantly up-regulation of adhesion, proliferation and differentiation. Moreover, the enhanced cell spreading morphology with decreased circularity and increased aspect ratio has demonstrated to improve osteogenic differentiation (Fig. 2b) [20]. These results are consistent with the published literatures regarding the little difference of osteogenic differentiation between normal clinical titanium and substrates fabricated by using 3D printing technology. These might be because of cells are hard to perceive the macro-scaled features fabricated using the current 3D printing technology. Therefore, further modifications of nanoscaled structures are always needed to engineer the micro/nano hierarchical topography [21]. However, the modification processes always simultaneously changed various material features (such as roughness, hardness, hydrophilicity, etc.), which are cell-sensitive characteristics [22–25]. In this study, the typical quasi-three-dimensional hierarchical topographies were controlled under the same processing, thus it was very helpful to undercover the mechanism underlying how cells response to the quasi-three-dimensional hierarchical topography.

**Fig. 7:**
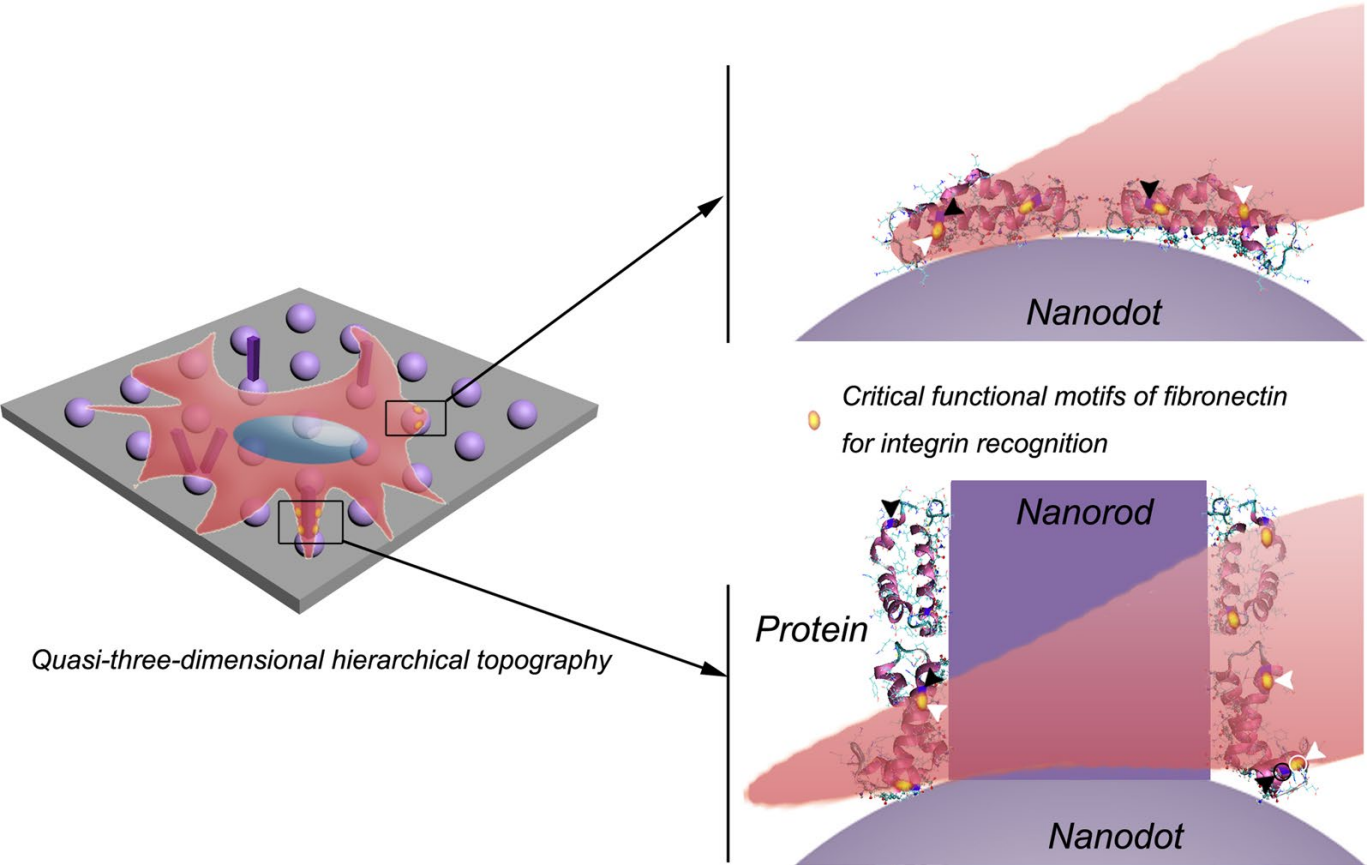
Schematic illustration of the possible mechanism underlying the enhanced osteogenesis of quasi-three-dimensional hierarchical topography. The distinct quasi-three-dimensional hierarchical topography is beneficial to the exposure of critical functional motifs of fibronectin, thus the cellular spreading into three-dimensional space, and eventually enhancing the osteogenesis

The initial protein adsorption onto material surface has been considered as the key role on the subsequent cellular responses [26, 27]. Therefore, we here focus on the adsorption behaviors of serum albumin and Fn, which are the major plasma protein and the critical functional glycoprotein of ECM proteins that binds to integrins, respectively [28, 29]. Although the total amount of initial adsorption of serum albumin on H-NR group was significantly up-regulated compared to other groups (Fig. 5a), cells seemed to more like to adhere on M-HR surface (Fig. 2c). These anti-intuitive results suggested that the amount of the preadsorbed serum albumin protein might be probably not the most important role to regulate the subsequent cellular adhesion. Further evaluation of the adsorption amount of Fn also indicated that there was no difference among various substrates (Fig. 5b). However, the total exposure amount as well as the relative exposure per Fn unit of HFN7.1 and mAb1937 critical functional motifs on M-NR were significantly up-regulated compared to that of other substrates. The HFN7.1 and mAb1937 motifs have been demonstrated to bind the 9th and 10th type III repeats of Fn involving cell adhesion domain (RGD) as well as the 8th type III domain closer to the synergy domain (PHSRN) of Fn, respectively [30]. Therefore, we here emphasized that the protein configuration but not the amount might be more critical to the cellular responses. And the distinct protein adsorption behaviors on quasi-three-dimensional hierarchical topography with moderate nanorods density, as schematically illustrated in Fig. 7, directly affected the strength of initial cell adhesion (Fig. 5c) as well as the subsequent proliferation, differentiation and osteointegration (Figs. 2, 3 and 6). And the up-regulated ratio of Ca:P on M-NR film group also suggested that CaP type might transferred from calcium-deficient apatite to hydroxyapatite (Fig. 3d, e) [31].

In addition to the bone tissue, epithelium and connective tissues are also able to form interfaces with the implant surface [32]. To further explore the fibrogenic response by fibroblast (the most common cell type in connective tissue) to produce the collagen nodular foci, we evaluated the adhesion, proliferation and collagen production behaviors of fibroblast on various substrates. The little difference of adhesion and proliferation between various substrates suggested fibroblast was not sensitive to the current quasi-three-dimensional hierarchical topography (Fig. 4a, b). However, they showed up-regulated collagen production potential on the normal titanium group compared to that of other substrates (Fig. 4c). These behaviors in difference with osteoblasts might be attributed to the distinct responses of certain cell types to different surface features. It is known that osteoblasts prefer rough surfaces, while fibroblasts favor smooth surfaces [33]. The underlying mechanism based on the initial protein adsorption configuration corresponding to the different integrin populations present to osteoblast and fibroblast still needs to be further investigated. In all, our results indicated that the current quasi-three-dimensional hierarchical topography is more suitable to form interface with bone tissue.

## Conclusion

In summary, we demonstrated the enhanced osteogenesis of distinct quasi-three-dimensional hierarchical topography composed of density-controlled titania nanodots and nanorods. We demonstrated that cellular viability of osteoblast but not fibroblast, such as adhesion, proliferation, differentiation was dependent on the density of nanorods. The middle density nanorods film (M-NR film) with 30 nm of dimension, 420 nm of height and 300 nm of spacing was identified to be the optimal characteristics for cellular pseudopods perception the distinct quasi-three-dimensional hierarchical topography. Histological analysis showed that the designed topography significantly enhanced new bone formation and the relative bone-to-implant contact after implantation. Strikingly, the biomechanical test in a rabbit tibia model revealed that the strength of bone integration of the quasi-three-dimensional hierarchical topography was more than 80% greater than that of the clinical titanium. This work, therefore, provides insights into the mechanism of cell-biomaterial interactions as well as a strategy for optimizing osteointegration of hard tissue coatings.
